# Supplementary material for: COVID-19 and Influenza Coinfection Outcomes among Hospitalized Patients in the United States: A Propensity Matched Analysis of National Inpatient Sample
Source: Vaccines (Basel). 2022 Dec 15;10(12):2159. doi: 10.3390/vaccines10122159 (PMC9783554; doi:10.3390/vaccines10122159)

Supplemental Files

Supplementary table:1	
**Variable**	**ICD-10 CM code**
Influenza A & B	J10.XX, J11.XX, J09.XX
Covid-19	U071, U00, U49, U50, U85, J1282
AKI	N17.XX, N99.0
Stroke	I63.XX, I60.XX, I61.XX, G43.6XX, I97.82XX, I97.81XX
Cardiac arrest	I46.XX, I49.0XX, I97.12XX, I97.71XX
Smoking	F17.XX, Z87.891
CAD	I25.10, I25.11, I25.118, I25.119, I252, I253, I25.4XX, I25.5, I25.6, I25.8XX, I25.7XX, I25.9XX
CHF, HTN, DM, Renal failure, Chronic pulmonary disease, Obesity	Elixhauser comorbidities were used
**Variable**	**ICD-10 procedure code**
Intubation	5A1945Z, 5A1955Z, 5A1935Z, 5A09357, 5A09457, 5A09557
Vasopressor use	3E030XZ, 3E033XZ, 3E040XZ, 3E043XZ, 3E050XZ, 3E053XZ, 3E060XZ, 3E063XZ
Hemodialysis	5A1D70Z, 5A1D90Z, 5A1D80Z, 5A1D00Z, 5A1D60Z

Supplemental Figure S1: Standardized bias across matched covariates.

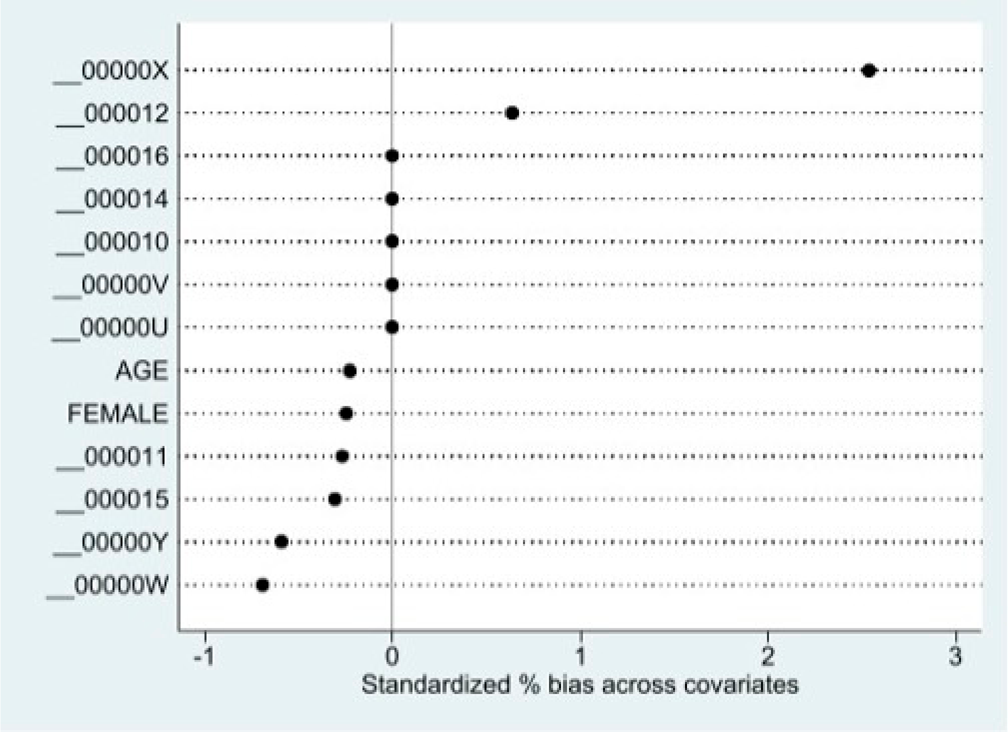

Supplemental Figure S2: Propensity score between Influenza positive and Influenza negative groups.

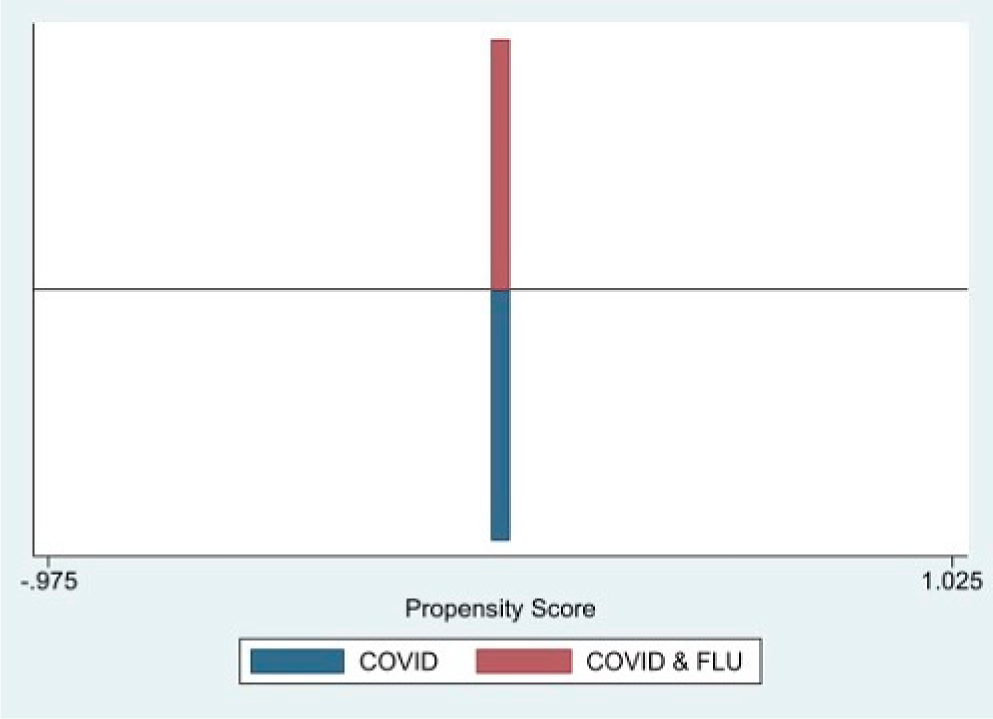

Supplement: Supplementary file 1 [file vaccines-10-02159-s001.zip › vaccines-2020555-supplementary.pdf]
